# Perceptions and priorities of perioperative staff and the public for sustainable surgery: a validated questionnaire study

**DOI:** 10.1097/MS9.0000000000000289

**Published:** 2023-05-03

**Authors:** Nishita Gadi, Kyle Lam, Amish Acharya, Jasmine Winter Beatty, Sanjay Purkayastha

**Affiliations:** Department of Surgery and Cancer, St Mary’s Hospital, London, UK

**Keywords:** carbon neutral, perioperative care, surgical care, sustainability

## Abstract

Surgery is a substantial contributor to healthcare-related emissions. Despite a drive to improve sustainability, few interventions have been adopted. Stakeholder engagement is considered a key barrier to implementation. This study aimed to determine the attitudes and beliefs of the perioperative staff and the public regarding sustainability initiatives in surgery, and whether differences exist between the two groups.

**Materials and Methods:**

Separate validated healthcare professional and public questionnaires were developed using a stepwise process. A systematic review was undertaken using Medline, Embase and Cochrane to identify key domains pertaining to sustainability and ensure content validity. Initial questionnaires were developed and refined using an iterative process of feedback from focus groups. Psychometric validation was conducted to remove question ambiguity. The final validated questionnaire was distributed to perioperative staff and the public using a multimodal approach involving online tools and in person.

**Results:**

Only 37.1% of perioperative staff reported the implementation of sustainability initiatives in their departments. Yet, staff (45.7%) and the public (48.2%) somewhat agreed that sustainability should influence a surgeon’s procedural decision-making. Insufficient staff education regarding sustainability was a potential cause for the lack of adoption, with 71.4% reporting they had no formal training. Moreover, discrepancies in the perceived importance of sustainability may have contributed. Staff and the public agreed that outcomes (38.6 vs. 42.7%, *P*=0.767) and surgeon experience with a technique (28.6 vs. 40.0%, *P*=0.082) were more important than sustainability. However, 40.9% of the public did not consider operative time an important factor compared to sustainability, while 45.7% of staff would only tolerate procedures 25% longer.

**Conclusions:**

Engaging stakeholders is central to implementing long-term environmentally sustainable initiatives in surgery without compromising patient outcomes. More work is needed to understand the relative trade-offs considered by perioperative staff and the public, as well as provide both groups with more pertinent education on ecological outcomes.

## Introduction

Highlights

- Improving environmental sustainability is a high priority for perioperative staff and the public.
- Few initiatives have currently been implemented into practice.
- A lack of education for all members of the surgical team is a significant barrier to environmental sustainability.
- An appreciation of the trade-offs used in surgical decision-making may also contribute to the adoption of novel initiatives.

The Lancet’s Commission on health and climate change stated that ‘climate change was the greatest threat to global health of the 21st century’^1^. Healthcare contributes substantially to this global carbon footprint. For instance, the U.K.’s National Health Service (NHS) is responsible for over a third of the U.K.’s public sector emissions and has a carbon footprint of 25 million tonnes of CO2e per year^2,3^. Across the globe, healthcare systems have recognised and reacted to this emergency; among these, the NHS was the first to set ambitious targets to reach Net Zero emissions by 2040^4^.

Surgery is a highly resource-intensive field within healthcare. It requires extensive sterilisation, generates large waste volumes and utilises significant amounts of energy^5^. Hospitals and surgical teams have therefore proposed multiple interventions for more sustainable surgery, ranging from the use of alternative anaesthetic agents to reusable surgical equipment and reduction of waste and energy inefficiencies within the theatre^6–8^. The primary focus of the literature surrounding these interventions has tended to be the environmental effectiveness of these interventions. While this is of course crucial to establish, we must also recognise that successful adoption and wide-scale uptake of these interventions is multifactorial, and therefore environmental effectiveness cannot be the sole consideration. The fact that widespread implementation of these interventions within surgical departments is yet to be achieved^8–10^, despite some strong evidence of positive environmental impacts, demonstrates the need to consider other factors which influence uptake.

A lack of engagement from all stakeholders, including members of perioperative staff and patients, has previously been shown to be a barrier to uptake^10–13^. Therefore, to achieve successful translation of sustainable interventions, we must first understand the perspectives of stakeholders who we aim to engage. Indeed, efforts have already been made to understand the views of perioperative staff concerning sustainability in surgery^10–13^. However, these studies have focused only on participants’ concerns around climate change and their willingness to engage in sustainable interventions. The reality of adopting sustainable practices into clinical practice is more complex. To gain a true picture of support for sustainable interventions amongst surgical teams, it is vital to understand how sustainability fits into the surgical decision-making framework and how staff believes it should be balanced with factors such as surgeon familiarity with a technique, patient outcomes and cost^14^. In parallel, we must examine patients’ views of the importance and the acceptability of sustainable interventions in a surgical context and their appetite for information on what practices have been adopted.

Therefore, this study aimed to determine the perspectives and priorities of perioperative staff and members of the public concerning sustainability in surgery and any differences that may exist between the two groups through the design and distribution of a novel validated questionnaire. Through this questionnaire study, we aimed to determine to what extent sustainability is a priority in the surgical decision-making framework and the key barriers to the future implementation of sustainable interventions. This will strengthen the evidence on how to implement these interventions so that more sustainable surgery can be achieved, Supplemental Digital Content 1, http://links.lww.com/MS9/A92.

## Methods

### Questionnaire design

**Figure 1:**
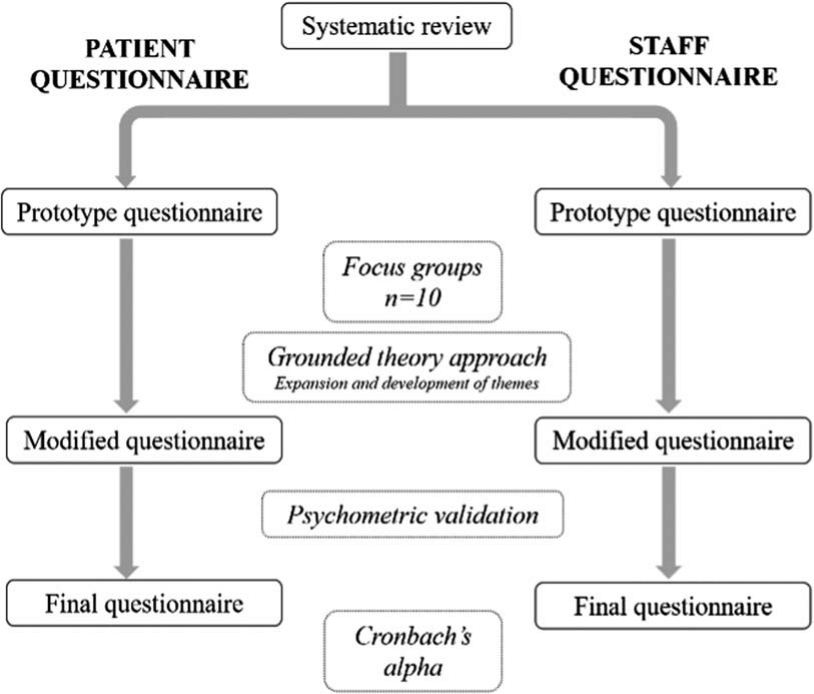
Diagram representing the development and validation of both patient and staff questionnaires.

A stepwise approach was used in this prospective, cross-sectional study to develop and validate a novel questionnaire exploring perspectives around sustainability in surgery based on a previously published methodology by John *et al* ^15^. The structure of this stepwise process is shown in Figure 1. Firstly, a systematic review was undertaken to determine key domains and to ensure the content validity of the questionnaire. A comprehensive literature search was performed on the Medline, Embase and Cochrane databases in consultation with a librarian at the British Medical Association library. Search terms included ‘Sustainability’, ‘Carbon footprint’, ‘Surgery’, ‘interventions’, ‘minimise’, and their synonyms. Free-text words were combined using Boolean operators in addition to medical subject heading (MeSH) terms. The full search strategy can be found in Supplementary Material A, Supplemental Digital Content 2, http://links.lww.com/MS9/A93. Themes from the literature were expanded upon to create two prototype questionnaires: one for healthcare staff and one for members of the public. Focus groups of both surgeons and members of the public (*n*=10) were conducted to determine feedback, improve prototype questionnaires, and further expand upon themes from the literature to ensure content validity. Members of the public provided a critical review of the materials to ensure the readability and acceptability of the questions. Psychometric validation was conducted to identify ambiguous questions which were subsequently reworded or omitted. Finally, Cronbach *α*’s test was calculated to assess the internal consistency and reliability of the questionnaire.

The final version of the two questionnaires can be found in Supplementary Material B, Supplemental Digital Content 2, http://links.lww.com/MS9/A93 and Supplementary Material C, Supplemental Digital Content 2, http://links.lww.com/MS9/A93. Questions were organised into three main domains: knowledge; priorities; and perceptions. The questionnaire consisted of multiple question types including multiple-choice, Likert-scale, sliding-scale, and free-text questions. Questions were worded both positively and negatively to prevent response acquiescence.

### Questionnaire distribution

Questionnaires were distributed to two groups. One questionnaire designed for healthcare staff was distributed to healthcare workers working in perioperative care including surgeons, anaesthetists, nurses and operating department practitioners. A second questionnaire was distributed to members of the public. Perioperative staff were recruited via e-mail or in person to participate. Members of the public were mainly recruited via the VOICE platform (https:// www.voice-global.org/), a patient and public involvement and engagement (PPIE) facilitating platform. Other public distribution channels included in person approaches and nontraditional methods, such as sustainability groups and social media. Members of the public were asked not only to complete the online questionnaire, but provide free-text feedback on the relevance of the questions or if areas had been missed, in order to iteratively improve questioning. No financial or other incentive was offered to participants. All patients were aged over 18 and based in the United Kingdom. No restrictions were placed upon geography, language or previous healthcare utility. Questionnaires were completed electronically via the Qualtrics XM Platform (Qualtrics) over a 4-week period between April and May 2022.

### Statistical analysis

Quantitative analysis was performed using GraphPad Prism version 9.3.0 for Mac OS. Routine descriptive statistics (counts and percentages) were performed, and chi-squared (*χ* ^2^) tests were used to determine statistically significant differences between respondents’ views. Statistical significance was defined as *P*<0.05. No sample size was conducted as this was an exploratory study on the perceptions of the public and surgical staff.

### Qualitative analysis

Free-text answers were thematically analysed using NVivo qualitative data analysis software (QSR International Pty Ltd. Version 12, 2018). After familiarisation with all free-text responses, one researcher (N.G.) identified potential codes though recurring concepts. Responses were subsequently independently coded by one researcher (N.G.) and validated by a second researcher (K.L.) and frequencies of codes were summarised.

### Ethical considerations

Full ethical approval was granted by the Institutional Review Board at Imperial College London, following review of the study protocol. A formal ethical review was deemed unnecessary due to the low-risk nature of the study. Informed consent was provided electronically by all participants prior to participation in the study. The study was conducted according to Strengthening the Reporting of cohort, cross-sectional and case–control studies in Surgery (STROCCS) guidance^16^. The study was prospectively registered on the Open Source Framework (DOI 10.17605/OSF.IO/E5CNJ) available online: https://osf.io/e5cnj/?view_only=b35c0d62749d4bbfb0316c26e6bdd523. It has also been registered on the Research Registry (UIN researchregistry8611) https://www.researchregistry.com/register-now#home/registrationdetails/63b069990351b60012e8ce94/.

## Results

### Participant demographics

Seventy perioperative staff members and 110 members of the public completed the questionnaire. Demographic data for all 180 participants are shown in Table 1. The majority of perioperative staff completing the questionnaire were male (40/70, 57.1%) and aged between 30 and 39 (33/70, 47.1%) while the majority of public participants were female (74/110, 67.3%) and aged between 18 and 29 (42/110, 38.2%). Perioperative staff respondents represented a variety of specialties, the most common of which were general surgery (21/70, 30%) and orthopaedic and trauma surgery (15/70, 21.4%). In all, 179/180 (99.4%) total respondents reported making at least one sustainable change in their personal life.

**Table 1:** Demographic data of participants

	Public, *N* (%)	Staff, *N* (%)	*χ* ^2^ | df	*P*
Total	110 (100)	70 (100)		
Sex				
Male	33 (30.0)	40 (57.1)	14.09 | 3	**0.0028**
Female	74 (67.3)	30 (42.9)		
Non-binary/third gender	2 (1.80)	0		
Prefer not to say	1 (0.90)	0		
Age				
18–29	42 (38.2)	8 (11.4)	46.45 | 6	**<0.0001**
30–39	9 (8.20)	33 (47.1)		
40–49	22 (20.0)	14 (20.0)		
50–59	17 (15.4)	12 (17.1)		
60–69	11 (10.0)	3 (4.29)		
70–79	7 (6.40)	0		
80+	2 (1.80)	0		
Perioperative specialty				
General surgery and subspecialties	–	21 (30.0)		
Orthopaedic and trauma surgery	–	15 (21.4)		
Anaesthetics	–	10 (14.3)		
Theatre nurse	–	7 (10.0)		
Not in specialty training yet	–	5 (7.14)		
Vascular surgery	–	4 (5.71)		
ENT surgery	–	1 (1.43)		
Gynaecological surgery	–	1 (1.43)		
Operating department practitioner	–	1 (1.43)		
Ophthalmology	–	1 (1.43)		
Plastic surgery	–	1 (1.43)		
Other	–	3 (4.29)		
Stage of training				
Foundation year	–	2 (2.86)		
Core training	–	6 (8.57)		
Specialty registrar	–	19 (27.1)		
SAS/associate specialist	–	5 (7.14)		
Consultant	–	22 (31.4)		
Nontraining post	–	6 (8.57)		
Other	–	10 (14.3)		
Personal sustainable changes				
Brought your own bags when shopping	102 (92.7)	65 (92.9)	5.78 | 13	0.953
Regularly practice recycling	93 (84.5)	66 (94.3)		
Switched off lights and appliances to save energy	93 (84.5)	63 (90.0)		
Reduced use of single-use/disposable items	78 (70.9)	62 (88.6)		
Bought second-hand items	57 (51.8)	37 (52.6)		
Increased use of public/active transport	54 (49.1)	42 (60.0)		
Bought produce that is grown locally or in season	53 (48.2)	37 (52.6)		
Changed your diet for sustainability	46 (41.8)	41 (58.6)		
Bought products with an environmental label	43 (39.1)	33 (47.1)		
Taken shorter showers to save water or energy	38 (34.5)	29 (41.4)		
Participated in environmental volunteering or campaigning	23 (20.9)	16 (22.9)		
Chosen to use peat-free compost	22 (20.0)	22 (31.4)		
Other	9 (9.10)	11 (15.7)		
None	1 (0.82)	0		

Statistical significance (*P* < 0.05) values are in bold.

ENT, ear, nose and throat; *N*, number; SAS, specialty doctors and associate specialist.

### Participant knowledge

Participant knowledge of environmental sustainability was tested through four questions. Firstly, participants were asked about their understanding of the term sustainability. Common responses centred around ‘environmental harm’ (*n*=54) and ‘stopping resource depletion’ (*n*=37) (Supplementary Material D, Supplemental Digital Content 2, http://links.lww.com/MS9/A93). Both the public and staff were asked about their knowledge of the NHS’s CO_2_ emissions (3) (Supplementary Material E, Supplemental Digital Content 2, http://links.lww.com/MS9/A93) and the volume of CO_2_ emissions from an average operation in the U.K. (57) (Supplementary Material E, Supplemental Digital Content 2, http://links.lww.com/MS9/A93) to test knowledge surrounding sustainable surgery. Both participant groups showed high variability in estimates, although staff participants’ estimates were closer to the true value in both instances. A significantly higher proportion of staff (36/70, 51.4%) compared to the public (16/110, 14.5%) stated they were aware of the environmental targets placed on the NHS. A higher proportion of staff also believed surgery has a negative environmental impact (52/70, 74.3% vs. 76/110, 69.1%).

### Participant’s current sustainable practices

**Figure 2:**
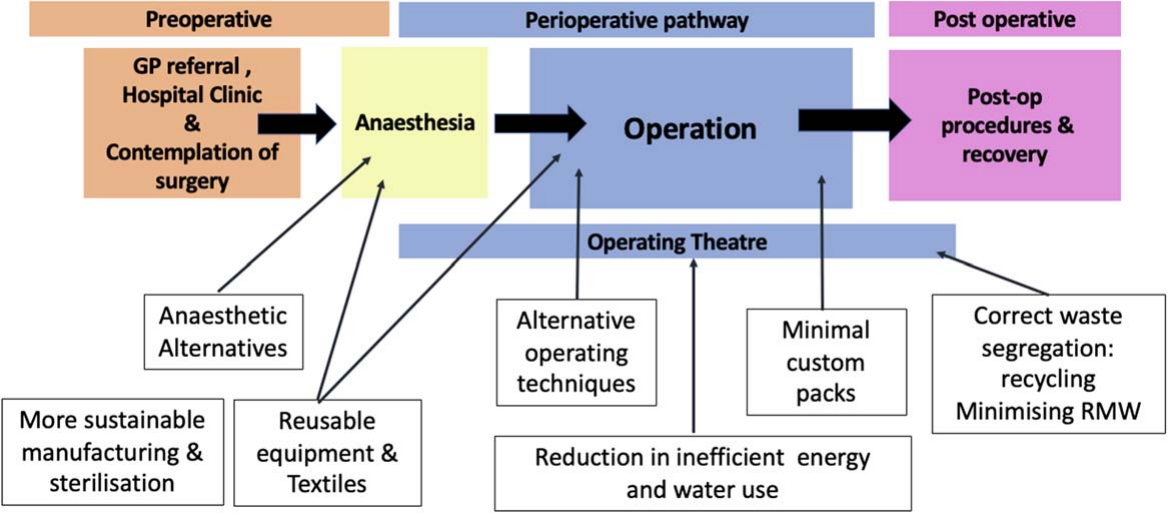
Schematic diagram illustrating how current and future sustainable interventions can be implemented in the surgical pathway. GP, General Practitioner.

The majority of staff participants agreed or strongly agreed that they were concerned about the threat of climate change and ecological emergency (63/70, 90.0%), with 60/70 (85.7%) reporting changes in behaviour in their personal life (Table 1). Staff members were asked about current sustainable practices within their surgical departments (Table 2). 71.4% (50/70) reported receiving no education on environmental sustainability. Waste-related initiatives, such as waste segregation (26/70, 37.1%) and minimising waste (25/70, 35.1%), were the most commonly reported sustainable interventions implemented. Respondents were also asked about their knowledge of existing sustainable interventions and possible future interventions in surgery (Fig. 2). A significant proportion (38.6%) of these related to management and organisational practices such as ‘mandating sustainable practice in the National Institute for Health and Care Excellence (NICE)’ and ‘giving resources for the implementation of sustainable practices’. The willingness of the public to accept particular sustainability interventions is shown in Supplementary Material F, Supplemental Digital Content 2, http://links.lww.com/MS9/A93.

**Table 2:** Current sustainable practices among perioperative staff

	*N* (%)
Has your surgical department implemented any changes to improve sustainability?	
Yes	26 (37.1)
No	19 (27.1)
Not sure	25 (35.7)
Have you received education or training on environmental sustainability in the workplace?	
Received training from my departments	5 (7.14)
Received training from hospital teaching	4 (5.71)
Received training from the deanery	0
Received training from Health Education England or other external body	2 (2.86)
Other	9 (12.9)
None	50 (71.4)
What does your surgical team do to actively implement sustainability initiatives into your practice?	
Recycle and correct waste segregation	26 (37.1)
Eliminate single-use items	5 (7.14)
Switch to reusable equipment that works in the same way	18 (25.7)
Minimise waste (e.g. not opening disposable equipment that is not needed)	25 (35.1)
Use a more sustainable anaesthetic agent	16 (22.9)
Use local/regional rather than general anaesthetic	15 (21.4)
Reduce heating, lighting, and water usage in empty operating theatres	12 (17.1)
Improve capacity/efficient uses of theatres	11 (15.7)
Other	2 (2.86)
None	21 (30.0)

### Participant priorities and perceptions

Both public and staff respondents were asked to balance the importance of sustainability against four other key factors within the surgical decision-making process: patient outcomes, financial cost, surgeon experience, and duration of surgery (Table 3). Of these factors, only procedure duration was found to be significantly different between the two cohorts. 40.9% (45/110) of the public stated that the duration of the procedure did not matter to them, and 27.7% (25/70) would be happy with a more sustainable procedure that was double the time of a less sustainable equivalent. Conversely, only 4.29% (3/70) of perioperative staff felt procedure duration was not important, with 25.7% (18/70) not accepting any prolongation to procedure duration (Table 3).

**Table 3:** Prioritisation of sustainability to other factors in the surgical decision-making process

	Public, *N* (%)	Staff, *N* (%)	*χ* ^2^ | df	*P*
Patient outcomes vs. sustainability				
Patient outcomes are much more important	47 (42.7)	27 (38.6)	1.83 | 4	0.767
Patient outcomes are more important	32 (29.0)	19 (27.1)		
Equally important	22 (20.0)	19 (27.1)		
Sustainability is more important	3 (2.73)	2 (2.86)		
Sustainability is much more important	1 (0.91)	0		
Financial cost to hospital/NHS vs. sustainability				
Financial cost to hospital is much more important	12 (10.9)	6 (8.57)	3.97 | 4	0.411
Financial cost to hospital is more important	11 (10.0)	10 (14.3)		
Equally important	50 (45.5)	28 (40.0)		
Sustainability is more important	26 (23.6)	16 (22.9)		
Sustainability is much more important	5 (4.55)	8 (11.4)		
Surgeon experience with technique vs. sustainability				
Surgeon experience with technique is much more important	44 (40.0)	20 (28.6)	8.27 | 4	0.082
Surgeon experience technique is more important	30 (27.3)	19 (27.1)		
Equally important	18 (16.4)	18 (25.7)		
Sustainability is more important	10 (9.09)	7 (10.0)		
Sustainability much more is important	1 (0.91)	5 (7.14)		
How much longer would you be happy for an operation to last if it were a more sustainable operation?				
0% longer	14 (12.7)	18 (25.7)	63.8 | 5	<0.0001
25% longer	16 (14.5)	32 (45.7)		
50% longer	1 (0.91)	9 (12.9)		
75% longer	1 (0.91)	3 (4.29)		
100% longer	25 (22.7)	3 (4.29)		
I don’t care about the duration of the procedure	45 (40.9)	3 (4.29)		
More environmentally sustainable initiatives should be chosen, even if it might cause an increased cost to the hospital				
Strongly agree	26 (23.6)	21 (30.0)	2.46 | 4	0.652
Somewhat agree	44 (40.0)	29 (41.4)		
Neither agree nor disagree	20 (18.2)	9 (12.9)		
Somewhat disagree	8 (7.27)	7 (10.0)		
Strongly disagree	3 (2.73)	4 (5.71)		
More environmentally sustainable initiatives should be chosen, even if it might cause a slight increase in risk of minor complications				
Strongly agree	10 (9.09)	15 (21.4)	6.22 | 4	0.184
Somewhat agree	22 (20.0)	18 (25.7)		
Neither agree nor disagree	15 (13.6)	9 (12.9)		
Somewhat disagree	24 (21.8)	14 (20.0)		
Strongly disagree	31 (28.1)	14 (20.0)		
How high a priority should spending on sustainability be for the NHS?				
Very high priority	17 (15.5)	22 (31.4)	7.61 | 5	0.107
High priority	59 (53.6)	33 (47.1)		
Neither high nor low priority	24 (21.8)	9 (12.9)		
Low priority	3 (2.73)	3 (4.29)		
Very low priority	1 (0.91)	1 (1.42)		

*N*, number; NHS, National Health Service.

Both public and staff respondents (79/110, 71.8% and 46/70, 65.7%, respectively) believed patient outcomes were more important than sustainability. Similarly, both the public and staff thought surgeon experience with the technique was more important than sustainability (74/110, 67.3% and 39/70, 55.7%). The most common response for both the public (50/110, 45.5%) and staff (28/70, 40%) was that financial cost is of equal importance to sustainability. Importantly, both the majority of the public (76/110, 69.1%) and staff (55/70, 78.6%) thought that sustainability should be a high spending priority for the NHS. Both public and staff participants agreed that sustainability should influence a surgeon’s choice of equipment and that sustainable choices should be chosen despite increased financial costs.

However, the majority of participants within both cohorts agreed that more environmentally sustainable alternatives should not be chosen if there was a potentially higher risk of minor complications (Table 3). Finally, participants were asked to state perceived barriers to improving sustainable surgical practice. Of these, the three most common were organisational factors in the hospital (127/180, 70.6%), the financial cost (142/180, 78.9%), and the time and effort required (137/180, 76.1%) (Table 4).

**Table 4:** Perspectives surrounding sustainability in surgery

	Public, *N* (%)	Staff, *N* (%)	*χ* ^2^ | df	*P*
Do you think surgery has a negative environmental impact?				
Definitely not	4 (3.64)	2 (2.86)	29.3 | 4	<0.0001
Probably not	15 (13.6)	5 (4.29)		
Might or might not	32 (29.1)	10 (14.3)		
Probably yes	35 (31.8)	11 (15.7)		
Definitely yes	21 (19.1)	41 (58.6)		
Sustainability should influence a surgeon’s choice of surgery or equipment				
Strongly agree	25 (22.7)	24 (34.3)	4.04 | 4	0.401
Somewhat agree	53 (48.2)	32 (45.7)		
Neither agree nor disagree	17 (15.5)	9 (12.9)		
Somewhat disagree	10 (9.09)	3 (4.29)		
Strongly disagree	5 (4.55)	2 (2.87)		
Patients should receive information about the sustainability of their operation				
Strongly agree	44 (40.0)	27 (38.6)	3.12 | 4	0.539
Somewhat agree	37 (33.6)	21 (30.0)		
Neither agree nor disagree	15 (13.6)	16 (22.9)		
Somewhat disagree	8 (7.27)	4 (5.71)		
Strongly disagree	6 (5.45)	2 (2.86)		
My choice of surgeon would be influenced by how sustainable their practice is, given patient outcomes are not affected				
Strongly agree	25 (22.7)	–	–	–
Somewhat agree	35 (31.8)	–		
Neither agree nor disagree	21 (19.1)	–		
Somewhat disagree	21 (19.1)	–		
Strongly disagree	8 (7.27)	–		
How much information do you think a patient should be given regarding the environmental impact of their surgery?				
None at all	16 (14.5)	11 (15.7)	1.45 | 4	0.835
Overall carbon footprint only if alternatives procedures are available to compare	43 (39.1)	26 (37.1)		
Overall carbon footprint even if alternatives procedures are not available	23 (20.9)	16 (22.9)		
Detailed breakdown of carbon footprint only if alternative procedures are available to compare	12 (10.9)	5 (7.14)		
Detailed breakdown of carbon footprint even if alternatives procedures are not available	11 (10.0)	10 (14.3)		
Patients should be able to make choices to increase the sustainability of their management				
Strongly agree	43 (61.4)	25 (35.7)	3.08 | 4	0.544
Somewhat agree	44 (62.9)	23 (32.9)		
Neither agree nor disagree	13 (18.6)	10 (14.3)		
Somewhat disagree	7 (10.0)	9 (12.9)		
Strongly disagree	3 (4.29)	3 (4.29)		
Hospitals should publish general information about the measures they are taking to reduce the carbon footprint of surgery				
Strongly agree	66 (57.3)	46 (65.7)	3.52 | 4	0.474
Somewhat agree	34 (30.9)	17 (24.3)		
Neither agree nor disagree	5 (4.55)	5 (7.14)		
Somewhat disagree	2 (1.81)	2 (2.86)		
Strongly disagree	3 (2.72)	0		
Surgeons should receive more education and training on sustainability in surgery				
Strongly agree	–	45 (64.3)		
Somewhat agree	–	14 (20.0)		
Neither agree nor disagree	–	8 (11.4)		
Somewhat disagree	–	3 (4.29)		
Strongly disagree	–	0		
What do you think are barriers to improving sustainability in surgery?				
Low priority to surgeons	67 (60.9)	51 (72.9)	16.5 | 8	0.035
Financial cost	85 (77.3)	57 (81.4)		
Time and effort it takes to implement	80 (72.7)	57 (82.4)		
Lack of awareness amongst surgeons	68 (61.8)	54 (77.1)		
Organisational factors in hospital	70 (63.6)	57 (81.4)		
Patients’ opinions	34 (30.9)	17 (24.3)		
Different priorities amongst industry	55 (50.0)	49 (70.0)		
Not sure	7 (6.36)	0		
Other	1 (0.91)	8 (11.4)		

## Discussion

Environmental sustainability is an increasing priority for healthcare systems globally. Surgery, due to its resource intensity, is considered one area where there is significant potential to improve carbon neutrality. However, surgery involves complex decision-making, and as this study has shown, the adoption of sustainable interventions has been slow, with only 37.1% of staff confirming changes in their units. The majority of these changes are related to nonoperative aspects such as correct waste segregation (37.1%) and minimising waste (35.1%). Operative changes such as incorporating reusable equipment (25.7%), moving to local anaesthetic (21.4%) and removing single-use devices (7.14%) were substantially less common. The extent of impact and success of these changes is unclear: there is likely variability in the degree of implementation, with some changes potentially being only partially implemented. Despite this, both the public (70.9%) and perioperative staff (80.0%) either strongly or somewhat agreed that sustainability should impact a surgeon’s choice of procedure. This influence was mitigated by factors including patient outcomes and surgical experience, which were identified by both groups as higher priorities than improved sustainability. Increasing the financial cost or the duration of the procedure, however, was considered acceptable to achieve greater sustainability. Appreciating these trade-offs is integral when implementing proposed sustainability initiatives into surgical practice and will help ensure effective interventions are adopted without compromising patient care

Improving the sustainability of surgical care has become a prime concern globally. In 2021, the Royal College of Surgeons of England (RCS) launched the ‘sustainability in surgery strategy’^17^, which outlines its means of adhering to a triple-bottom-line framework evaluating the social, financial, and environmental impacts of the practice. One of the core tenets of the RCS strategy is to empower clinicians to lead initiatives within their own practice and organisations. Despite staff being concerned about an emerging climate crisis, and a recognition of the ecological impact of surgery, few had noted real-world changes in clinical practice. Waste management was the most commonly cited area in which changes were noted, but this was still low at 37.1%. These nonoperative strategies can have a profound impact on emissions. Studies have estimated the carbon footprint associated with disposing of noninfectious offensive waste is more than four times lower than clinical waste due to the need for high-temperature incineration. Mischaracterising waste can therefore lead to avoidable emissions^18^. Furthermore, only a limited repertoire of interventions had been implemented. One reason for this is that currently, staff lack the necessary education to adopt these initiatives. Approximately 70% of staff reported they had received no formal training on sustainability. Furthermore, only 2.86% of respondents received teaching from Health Education England, the organisation responsible for the coordination of education for the health workforce. Similar findings have previously been reported in various medical specialties. Harris *et al*.^10^ demonstrated that 60% of surgeons felt they had inadequate training on environmental sustainability but noted that 51% felt they had sufficient knowledge to implement changes to practice. This group, however, only examined the perspective of surgeons, and not anaesthetists or nursing staff, who are integral to the perioperative pathway. The latter represents an important stakeholder, with studies showing that theatre nurses feel they cannot impact ecological activities, despite being involved in equipment procurement, theatre utilisation and waste management. There is, therefore, a need for a holistic approach to sustainability education, incorporating all perioperative healthcare workers. In addition, this study has highlighted that there is also a need to engage with hospital management, with both the public (63.6%) and staff (81.4%) reporting organisational barriers to the implementation of sustainability drives. Engagement with healthcare leadership will not only help to overcome logistical challenges in procurement and facilitating implementation but also drive sustainability education at the local level.

This education will also underpin the surgical decision-making paradigm. Shared decision-making is espoused as the gold standard in care; ensuring that stakeholders are unified in their priorities will help catalyse long-lasting changes. It is, therefore, important to note that both the public and perioperative staff agree that sustainability should influence surgical decision-making. This process, however, is multifaceted. Whilst both groups agreed about the importance of considerations such as patient outcomes, surgical expertise, and financial cost with respect to sustainability, there was a disparity regarding procedural length. Most public respondents did not consider operative duration a significant factor, and almost a quarter were willing to tolerate procedures that were twice as long to achieve environmental outcomes. On the other hand, most perioperative staff believed a maximum duration of 25% longer than a traditional technique was acceptable. This may represent a lack of public understanding regarding the risks of prolonged operative time and appreciation that longer operative times may lead to longer waiting lists, which could impact this tolerance of longer operations. This potential lack of understanding, however, further highlights the need for greater transparency in surgical decision-making. In addition, it may also demonstrate the differing perspectives of healthcare workers and patients on what is acceptable when achieving sustainability aims. Taking these factors in conjunction, it could be considered that the public is willing to undergo a more sustainable procedure which is twice as long, but not at the cost of worse outcomes or minor complications. Understanding these trade-offs is, therefore, crucial in safely implementing ecological initiatives in surgery. Central to this is the need for patients to be given adequate information to make an informed decision. According to this study, the granularity of this information needs only be at the level of carbon footprints of all available management alternatives. This transparency should not be limited to procedural information but include the measures taken by healthcare organisations to achieve environmental sustainability. This will facilitate shared decision-making, as well as help to tackle the organisational barriers to effective adoption currently seen.

These findings should be taken into consideration with the limitations of the study. Despite using a robust evidence synthesis and methodology to ensure the content and construct validity of the questionnaire, as a cross-sectional evaluation, the study is subject to selection and response biases. Although the latter were minimised using an anonymous online survey, there was potential for participants to alter responses in the initial focus groups or if they were recruited in person. Furthermore, this survey only represents a subset of viewpoints of both surgical staff and the public. Whilst incorporating both groups ensured a breadth of perspectives was attained, these cannot be considered generalisable. Participants enrolled may, for example, represent a more well-informed and/or more-interested subgroup or those with greater exposure to the challenges of environmental sustainability. An attempt was made to reduce this by not limiting recruitment to a particular institution or region, but this could not be guaranteed. It is also important to note that the majority of perioperative staff participants were surgeons; theatre nurses and operating department practitioners represented only a minority of perioperative staff participants. Having more nonsurgeon staff enrolled in the study would have increased the generalisability of the findings. As the operative workflow is dependent upon others, theatre nurses and operating department practitioners, greater inclusion may highlight more nuanced perspectives as to why certain initiatives, for example waste segregation, are not ubiquitously implemented and provide greater understanding of the impact of sustainability beyond the procedure itself. Moreover, the questioning could also focus on the fidelity of the implementation of current initiatives. It is possible that there was a greater motivation for sustainability drives, but these were poorly introduced. On the other hand, it may be the case that existing initiatives are only infrequently utilised. Though beyond the scope of this study, future examinations may look to determine the quality and adherence to current initiatives.

Future work should look to address these issues, as well as examine how perspectives differ geographically and by surgical specialty. This will help to better categorise the current barriers to the adoption of sustainability initiatives and whether they are context-dependent or systemic. Moreover, further work should critically evaluate the relative importance attributed to the different factors impacting surgical decision-making, including sustainability. This should occur across the breadth of the stakeholder group, including patients, perioperative staff, and management, to highlight which prospective initiatives are likely to be better accepted and potentially worth adopting. It will be particularly important to include representation from departmental and hospital leadership in future studies, as they undoubtedly have a substantial influence on local uptake of interventions. Moreover, the engagement of these groups will be crucial in addressing key barriers to implementation, such as cost, education, and interdepartmental factors.

## Conclusions

Improving the environmental sustainability of surgical care is a prime public health concern, with perioperative staff and the public both identifying it as a high priority. Despite this, few interventions have been implemented thus far, and those which have concern only a subsection of surgical practice. Underpinning this lack of adoption is a paucity of available training for perioperative staff, including from national health education organisations. Furthermore, understanding the acceptability of sustainability initiatives in relation to other determinants of decision-making is crucial to ensure the safe and effective implementation of environmental strategies in surgical care.

## Ethical approval

Full ethical approval was granted by Dr Nooreen Shaikh, Coordinator of Imperial College London’s Research Governance and Integrity Team (RGIT), following a review of the study protocol. A formal ethical review was deemed unnecessary due to the low-risk nature of the study.

## Author contribution

K.L., A.A., and S.P.: conceived and designed the study; K.L., N.G., J.W.B., and A.A.: designed and distributed the questionnaire and analysed the data; K.L., N.G., and A.A.: drafted the manuscript; J.W.B. and S.P.: contributed significant amendments to the final manuscript.

## Sources of funding

This study was unfunded; however, infrastructural support was provided by the NIHR Imperial Biomedical Research Centre (BRC).

## Conflicts of interest disclosure

The authors have no conflicts of interest to declare.

## Research registration unique identifying number (UIN)

1. Name of the registry: Research Registry.
2. Unique identifying number or registration ID: UIN researchregistry8611.
3. Hyperlink to your specific registration (must be publicly accessible and will be checked): https://www.researchregistry.com/registernow#home/registrationdetails/63b069990351b60012e8ce94/.

## Guarantor

Kyle Lam.

## Provenance and peer review

Not commissioned, externally peer-reviewed.

## Supplementary Material
