# Supplementary material for: Perceptions and priorities of perioperative staff and the public for sustainable surgery: a validated questionnaire study
Source: Ann Med Surg (Lond). 2023 May 3;85(6):2400–8. doi: 10.1097/MS9.0000000000000289 (PMC10289730; doi:10.1097/MS9.0000000000000289)
Supplement: Supplementary file 2 [file ms9-85-2400-s002.docx]

**SUPPLEMENTARY MATERIAL**

**A: Search Strategy**

Medline / EMBASE

1. “Conservation of Natural Resources”/ or Carbon Footprint/

2. (sustainability or “carrying capacity”).mp.

3. ((conserv$ or sustain$ or monitor$ or protect$ or preserv$ or restor$ or destruct$) adj (ecosystem or eco-system or natural resource$ or environment$ or ecolog$ or nature)).mp.

4. (climate adj (change or emergency or crisis or breakdown)).mp.

5. (“global warming” or “global heating”).mp.

6. (carbon adj (emission$ or footprint$ or reduc$)).mp.

7. (“greenhouse gas$” or “greenhouse effect$”).mp.

8. or/1-7

9. exp Surgical Procedures, Operative/

10. (surg$ or neurosurg$ or “surgical procedure$” or “surgical practice$”).mp.

11. su.fs.

12. (operating adj (theatre$ or theater$ or room or suite)).mp.

13. exp Surgeons/

14. (clinician$ or doctor$ or surgeon$ or physician$).mp.

15. or/9-14

16. 8 and 15

17. ((minimi#$ or reduc$ or lower$ or recyc$ or reus$ or decreas$ or improve$ or manag$ or interven$ or strateg$) adj10 surg$).mp.

18. (“carbon neutral$” or carbon-neutral or “carbon free” or carbon-free or “low carbon” or “net zero” or net-zero or “climate positiv$” or “green surg$” or “green energy”).mp.

19. (sustain$ adj4 surg$).mp.

20. ((NHS or “National health service”) adj2 (“sustainable development” or “sustainable healthcare” or greener or “net zero”)).mp.

21. (“best practice$” or “gold standard$”).mp.

22. or/17-21

23. 16 and 22

24. limit 23 to english language

25. limit 24 to last 10 years

Cochrane

1. [mh “Conservation of Natural Resources”] OR [mh “Carbon Footprint”]

2. (sustainability OR “carrying capacity”):ti,ab,kw

3. ((conserv* OR sustain* OR monitor* OR protect* OR preserv* OR restor* OR destruct*) NEAR/1 (ecosystem OR eco-system OR natural NEXT resource* OR environment* OR ecolog* OR nature)):ti,ab,kw

4. (climate NEAR/1 (change OR emergency OR crisis OR 122 breakdown)):ti,ab,kw

5. (“global warming” OR “global heating”):ti,ab,kw

6. (carbon NEAR/1 (emission* OR footprint* OR reduc*)):ti,ab,kw

7. (greenhouse NEXT gas* OR greenhouse NEXT effect$):ti,ab,kw

8. {OR #1-#7}

9. [mh “Surgical Procedures, Operative”]

10. (surg* OR neurosurg* OR surgical NEXT procedure* OR surgical NEXT practice*):ti,ab,kw

11. MeSH descriptor: [] explode all trees and with qualifier(s): [surgery - SU]

12. (operating NEAR/1 (theatre* OR theater* OR room OR suite)):ti,ab,kw

13. [mh Surgeons]

14. (clinician* OR doctor* OR surgeon* OR physician*):ti,ab,kw

15. {OR #9-#14}

16. #8 AND #15

17. ((minimis* OR minimiz* OR reduc* OR lower* OR recyc* OR reus* OR decreas* OR improve* OR manag* OR interven* OR strateg*) NEAR/10 surg*):ti,ab,kw

18. (carbon NEXT neutral* OR carbon-neutral OR “carbon free” OR carbon- 28 free OR “low carbon” OR “net zero” OR net-zero OR climate NEXT positiv* OR green NEXT surg* OR “green energy”):ti,ab,kw

19. (sustain* NEAR/4 surg*):ti,ab,kw

20. ((NHS OR “National health service”) NEAR/2 (“sustainable development” 3 OR “sustainable healthcare” OR greener OR “net zero”)):ti,ab,kw

21. (best NEXT practice* OR gold NEXT standard*):ti,ab,kw

22. {OR #17-#21}

23. #16 AND #22

**B: Public questionnaire**

1. Are you?

- Male

- Female

- Non binary/third-gender

- Prefer not to say

2. How old are you?

- 18-29

- 20-39

- 30-39

- 40-49

- 50-59

- 60-69

- 70-79

- 80+

3. What is your definition of environmental sustainability?

4. Do you think surgery has a negative environmental impact?

- Definitely not

- Probably not

- Might or might not

- Probably yes

- Definitely yes

5. What percentage of UK’s net CO2 emissions do you think the NHS is responsible for?

(Slider from 0-25%)

6. How much CO2 emissions do you think a single operation in the UK produces, on average? (Reference: A return flight from London to Paris produces approximately 114kg CO2e per passenger)

(Slider from 0-500kg CO2e)

7. Are you aware of the environmental targets which have been placed on the NHS?
- Yes

- No

*For the following questions, the definition of environmental sustainability is the likelihood that environmental resources will be protected and maintained for future generations.*

8. What interventions are you aware of that make surgery more environmentally sustainable or environmentally friendly?

9. Which sustainable changes have you made in your personal life?

- Regularly practice recycling

- Reduced use of single use/disposable items

- Increased use of public/active transport as opposed to driving

- Switched off lights and appliances to save energy/to protect the environment

- Brought your own bags when shopping

- Bought products with an environmental label (shows that a product or service is produced with less impact on the environment)

- Bought produce that is grown locally or in season

- Bought second-hand items

- Taken shorter showers to save water or energy

- Participated in environmental volunteering or campaigning

- Chosen to use peat free compost

- Changed your diet for sustainability (e.g. eat less meat and dairy)

- None

- Other

10. Have you undergone an operation in the last 2 years?

- Yes

- No

10b. Were you informed about the sustainability of the procedure you were undergoing

- Yes

- No

*DISCLAIMER: Sustainable care is often lower cost and can have fewer complications. However, in the next section items have been phrased specifically, to help us understand priorities better.*

11. Which is more important when choosing a procedure: patient outcomes or sustainability?

- Patient outcomes are much more important

- Patient outcomes are more important

- Equally important

- Sustainability is more important

- Sustainability is much more important

12. Which is more important when choosing a procedure: financial cost to the hospital/NHS or sustainability?

- Financial cost to hospital is much more important

- Financial cost to hospital is are more important

- Equally important

- Sustainability is more important

- Sustainability is much more important

13. If a more sustainable procedure increased the duration of a procedure, how much longer would you be happy for an operation to last if it were a more sustainable operation?

- 0% longer (I wouldn’t choose the sustainable procedure if it increases duration)

- 25% longer

- 50% longer

- 75% longer

- 100% longer (double the original duration)

- I don’t care about the duration of the procedures

14. Multiple techniques can be used for an operation eg keyhole surgery or open surgery. This question asks you which is more important when choosing a technique: the surgeon’s experience of the technique or the sustainability of the technique, given equal patient outcomes.

- Surgeon experience with technique is much more important

- Surgeon experience with technique is more important

- Equally important

- Sustainability is more important

- Sustainability is much more important

15. How high a priority should spending on sustainability be for the NHS?

- Very high priority

- High priority

- Neither high nor low priority

- Low priority

- Very low priority

16. Please rate how much you agree with the following statements.

a) Sustainability should influence a surgeon’s choice of surgery or equipment

b) Patients should receive information about the sustainability of their operation

c) Patients should be able to make choices to improve the sustainability of their management

d) My choice of surgeon would be influenced by how sustainable their practice is given patient outcomes are not affected

e) Hospitals should publish general information about the measures they are taking to reduce the carbon footprint of surgery

f) More environmentally sustainable initiatives should be chosen even if it might cause a slight increase in risk of minor complications

- Strongly agree

- Somewhat agree

- Neither agree nor disagree

- Somewhat disagree

- Strongly disagree

17. If you were to undergo an operation in the future, how much information about its sustainability would you like?

- None at all

- Overall carbon footprint only if alternative procedures are available to compare

- Overall carbon footprint even if alternative procedures are not available

- Detailed breakdown of carbon footprint only if alternative procedures are available to compare

- Detailed breakdown of carbon footprint even if alternative procedures are not available

18. Which of these would you be willing to accept in your operation to make it more sustainable?

*NOTE: for the last statement, a minor complication is a complication of surgery that can be treated at the bedside, and does not require further surgery.*

*(Surgically classified as Clavien-Dindo 1, more information can be found here [link provided])*

a) Recycling and correct waste segregation

b) Eliminating single-use items

c) Switching to reusable equipment that works in the same way

d) Minimising waste (e.g. not opening disposable equipment that is not needed)

e) Using a more sustainable anaesthetic agent (an anaesthetic agent produces a local or general loss of sensation such as pain)

f) Using local/regional rather than general anaesthesia if safe to do so (numbing a particular area of your body instead of being put to sleep)

g) Reducing heating, lighting and water usage in empty operating theatres

h) Improving capacity/efficient uses of theatres

i) Increased cost for the hospital due to a more environmentally sustainable initiative chosen

j) Slight increase in risk of minor complications of surgery due to a more environmentally sustainable initiative chosen

- Very willing

- Somewhat willing

- Neither willing or unwilling

- Somewhat unwilling

- Very unwilling

19. What do you think are barriers to improving sustainability in surgery?

- Low priority to surgeons

- Financial cost

- Time and effort it takes to implement

- Lack of awareness among surgeons

- Organisational factors in hospital

- Patients’ opinions

- Different priorities amongst industry

- Not sure

- Other

20. Can you think of any ways to improve sustainability in surgery?

**C: Perioperative staff questionnaire**

1. Are you?

- Male

- Female

- Non binary/third-gender

- Prefer not to say

2. How old are you?

- 18-29

- 20-39

- 30-39

- 40-49

- 50-59

- 60-69

- 70-79

- 80+

3. What stage of training are you in?

- Foundation Year

- Core surgical trainee/Internal medicine training

- Specialty Registrar

- SAS/Associate specialist

- Consultant

- Non training post

- Other

4. What specialty are you in?

- Anaesthetics

- ENT

- General surgery (and subspecialties)

- Ophthalmology

- Orthopaedics/Trauma surgery

- Plastic surgery

- Vascular surgery

- Not in specialty training yet

- Cardiothoracic surgery

- Urology

- Gynaecological surgery

- Theatre Nurse

- Operating Department practitioner

- Other

5. What is your definition of environmental sustainability?

6. Do you think surgery has a negative environmental impact?

- Definitely not

- Probably not

- Might or might not

- Probably yes

- Definitely yes

7. What percentage of UK’s net CO2 emissions do you think the NHS is responsible for?

(Slider from 0-25%)

8. How much CO2 emissions do you think a single operation in the UK produces, on average? *(Reference: A return flight from London to Paris produces approximately 114kg CO2e per passenger)*

(Slider from 0-500kg CO2e)

9. Are you aware of the environmental targets which have been placed on the NHS?
- Yes

- No

*For the following questions, the definition of environmental sustainability is the likelihood that environmental resources will be protected and maintained for future generations.*

10. What interventions are you aware of that make surgery more environmentally sustainable or environmentally friendly?

11. Please rate how much you agree with each of the following statements

a) I am concerned about the threat of climate change and ecological emergency

b) I am well informed about climate change and ecological emergency and the impact it has on the natural world

c) My concern about climate change has made me change my behaviour in my personal life

d) My concern about climate change has made me change my behaviour at work

e) Surgeons have a responsibility to be aware of the environmental impact of surgical services

- Strongly agree

- Somewhat agree

- Neither agree nor disagree

- Somewhat disagree

- Strongly disagree

12. Which sustainable changes have you made in your personal life?

- Regularly practice recycling

- Reduced use of single use/disposable items

- Increased use of public/active transport as opposed to driving

- Switched off lights and appliances to save energy/to protect the environment

- Brought your own bags when shopping

- Bought products with an environmental label (shows that a product or service is produced with less impact on the environment)

- Bought produce that is grown locally or in season

- Bought second-hand items

- Taken shorter showers to save water or energy

- Participated in environmental volunteering or campaigning

- Chosen to use peat free compost

- Changed your diet for sustainability (e.g. eat less meat and dairy)

- None

- Other

13. Has your surgical department implemented any changes to improve sustainability?

- Yes

- No

- Not sure

14. Have you ever received education or training on environmental sustainability in the workplace>

- None

- Received training from my departments

- Received training from hospital teaching

- Received training from deanery

- Received training from Health Education England or other external body

- Other

15. What do your surgical team do to actively implement sustainability initiatives into your practice?

- None

- Recycle and correct waste segregation

- Eliminate single-use items

- Switch to reusable equipment that works in the same way

- Minimise waste (e.g. not opening disposable equipment that is not needed)

- Use a more sustainable anaesthetic agent

- Use local/regional rather than general anaesthetic

- Reduce heating, lighting and water usage in empty operating theatres

- Improve capacity/efficient uses of theatres

- Other

*DISCLAIMER: Sustainable care is often lower cost and can have fewer complications. However, in the next section items have been phrased specifically, to help us understand priorities better.*

16. Which is more important when choosing a procedure: patient outcomes or sustainability?

- Patient outcomes are much more important

- Patient outcomes are more important

- Equally important

- Sustainability is more important

- Sustainability is much more important

17. Which is more important when choosing a procedure: financial cost to the hospital/NHS or sustainability?

- Financial cost to hospital is much more important

- Financial cost to hospital is are more important

- Equally important

- Sustainability is more important

- Sustainability is much more important

18. If a more sustainable procedure increased the duration of a procedure, how much longer would you be happy for an operation to last if it were a more sustainable operation?

- 0% longer (I wouldn’t choose the sustainable procedure if it increases duration)

- 25% longer

- 50% longer

- 75% longer

- 100% longer (double the original duration)

- I don’t care about the duration of the procedures

19. Multiple techniques can be used for an operation eg keyhole surgery or open surgery. This question asks you which is more important when choosing a technique: the surgeon’s experience of the technique or the sustainability of the technique, given equal patient outcomes.

- Surgeon experience with technique is much more important

- Surgeon experience with technique is more important

- Equally important

- Sustainability is more important

- Sustainability is much more important

20. How high a priority should spending on sustainability be for the NHS?

- Very high priority

- High priority

- Neither high nor low priority

- Low priority

- Very low priority

21. Please rate how much you agree with the following statements.

*NOTE: for the last statement, a minor complication is defined as a complication of surgery that can be treated at the bedside, e.g. with antiemetics, antipyretics, analgesics, diuretics and electrolytes, and does not require surgical, endoscopic or radiological intervention. (Surgically classified as Clavien-Dindo I, more information can be found here [link supplied].)*

a) Sustainability should influence a surgeon’s choice of surgery or equipment

b) Patients should receive information about the sustainability of their operation

c) Patients should be able to make choices to improve the sustainability of their management

d) My choice of surgeon would be influenced by how sustainable their practice is given patient outcomes are not affected

e) Hospitals should publish general information about the measures they are taking to reduce the carbon footprint of surgery

f) More environmentally sustainable initiatives should be chosen even if it might cause a slight increase in risk of minor complications

- Strongly agree

- Somewhat agree

- Neither agree nor disagree

- Somewhat disagree

- Strongly disagree

22. How much information do you think a patient should be given regarding the environmental impact of their surgery?

- None at all

- Overall carbon footprint only if alternative procedures are available to compare

- Overall carbon footprint even if alternative procedures are not available

- Detailed breakdown of carbon footprint only if alternative procedures are available to compare

- Detailed breakdown of carbon footprint even if alternative procedures are not available

23. What do you think are barriers to improving sustainability in surgery?

- Low priority to surgeons

- Financial cost

- Time and effort it takes to implement

- Lack of awareness among surgeons

- Organisational factors in hospital

- Patients’ opinions

- Different priorities amongst industry

- Not sure

- Other

24. Can you think of any ways to improve sustainability in surgery?

**D: Thematic analysis of participants’ definition of the term environmental sustainability**

| What is your definition of environmental sustainability? | Public frequency (n) | Staff frequency (n) | Example |
| --- | --- | --- | --- |
| No environmental harm | 34 | 20 | ‘Making sure that our actions and choices don’t harm the planet, and that we’ll be able to continue to do them in the future’ |
| Stop depletion of resources | 23 | 14 | ‘A system which can reuse and regenerate resources used’; ‘Using natural resources without depleting them for future generations’ |
| Preservation of environment for the future | 20 | 7 | ‘Environment being able to flourish and provide for us in long run’ |
| Initiatives that help the environment eg recycling | 19 | 6 | ‘Conserve, recycle resources for future generations’ |
| Reducing carbon emissions | 14 | 11 | ‘Reduce the carbon emissions of surgery’; ‘Net zero carbon footprint’ |
| Waste reduction and proper waste disposal | 10 | 4 | ‘Able to decrease waste’; ‘Doing things that will not generate landfill or excess pollution/waste, in a way that can be done repeatedly’ |
| Preserving ecosystems | 7 | 4 | ‘To allow us to sustain our ecosystems’ |
| Using renewable energy and materials | 4 | 11 | ‘Renewable energy and products’; ‘Maintaining the earth’s natural resources by using renewable materials where possible’ |
| Environmental protection | 4 | 6 | ‘Preserving resources and protecting ecosystems’ |
| In conjunction with good health and well-being | 4 | 2 | ‘Protect global ecosystems to support health and well-being’ |
| Public responsibility | 3 | 3 | ‘It is the responsibility…’; ‘Responsibility to conserve’ |
| Maintaining biodiversity | 3 | 2 | ‘Actions to improve planetary health, climate change and biodiversity’; ‘Protection humans and symbiotic species’ |
| Reduction in climate change and greenhouse gases | 3 | 2 | ‘The most important aspect is reducing climate change gases’ |
| Green living | 1 | 1 | ‘Try to be greener’ |

**E: Knowledge of participants concerning sustainability in surgery**


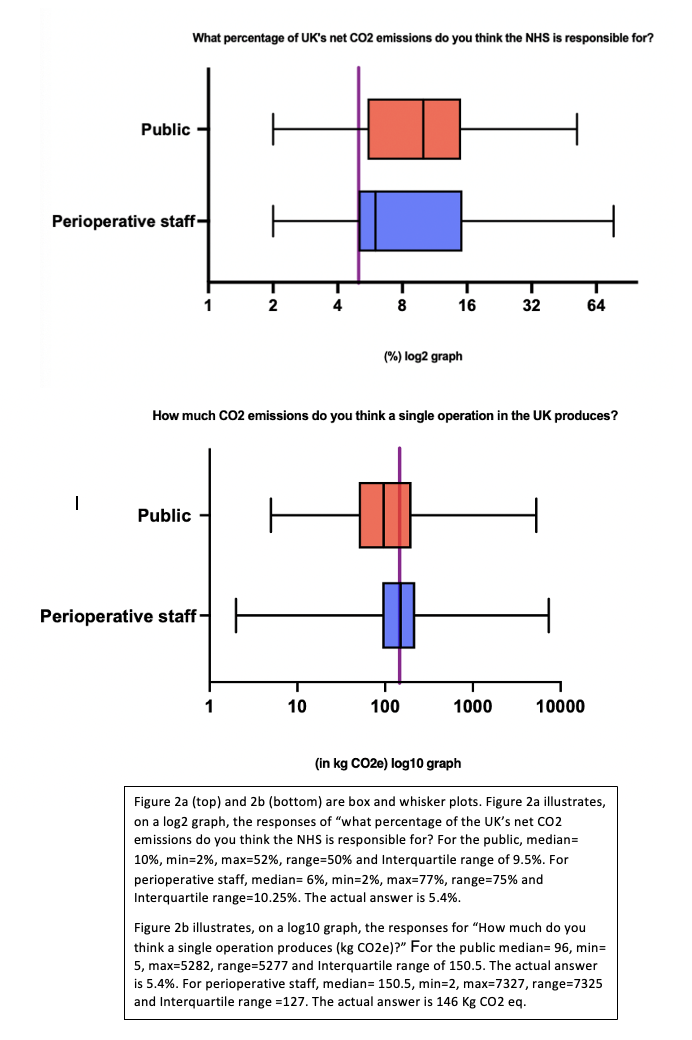


Figure: Box and whisker plots representing participant estimates concerning questions surrounding CO2 emissions from healthcare. The NHS is responsible for 5.4% of emissions^1^ (Public 10% (2%-52%); Staff 6% (2-77%). A single operation on average produces 146kg CO2e^2^ (Public 96kg (5kg-5282kg); Staff 150.5kg (2kg-7327kg)).

^1^ Wise J. Climate emergency: new expert panel will set out how NHS can achieve net zero BMJ 2020; 368 :m310 doi:10.1136/bmj.m310

^2^MacNeill A.J., Lillywhite R., Brown C.J., The impact of surgery on global climate: a carbon footprinting study of operating theatres in three health systems Lancet Planet Health. 2017 Dec;1(9):e381-e388. doi: 10.1016/S2542-5196(17)30162-6

**F: Public willingness to accept sustainable interventions**

**
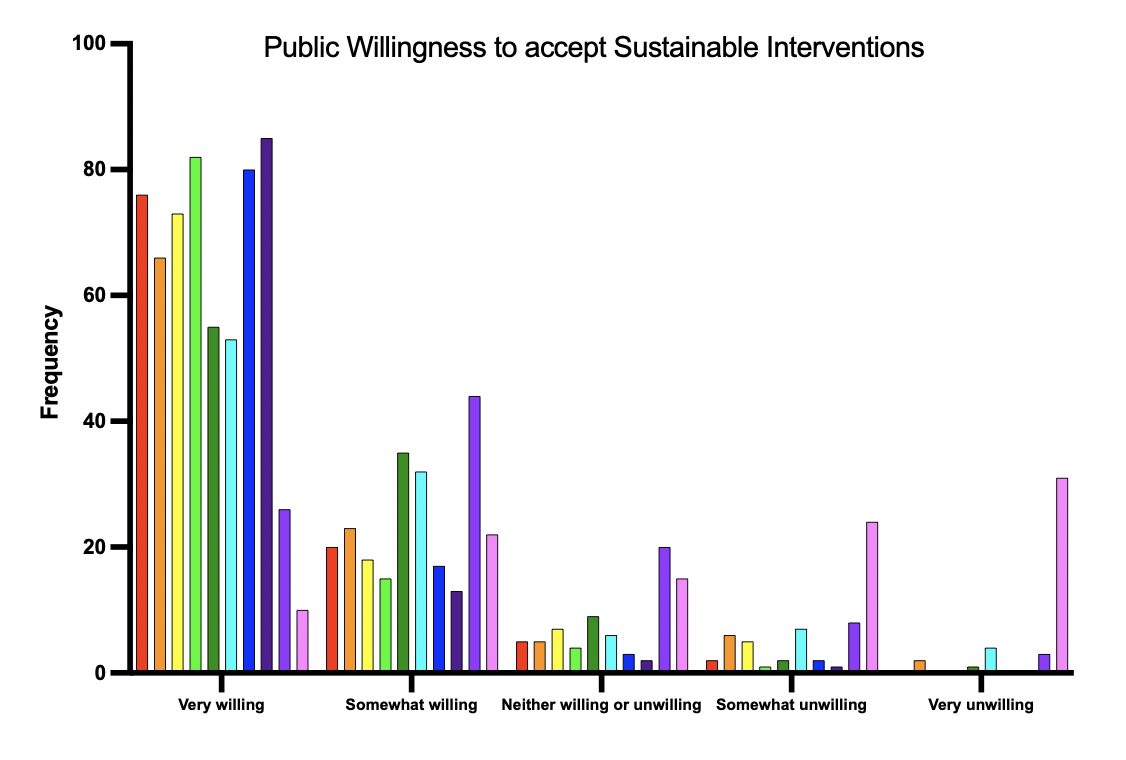
**

**
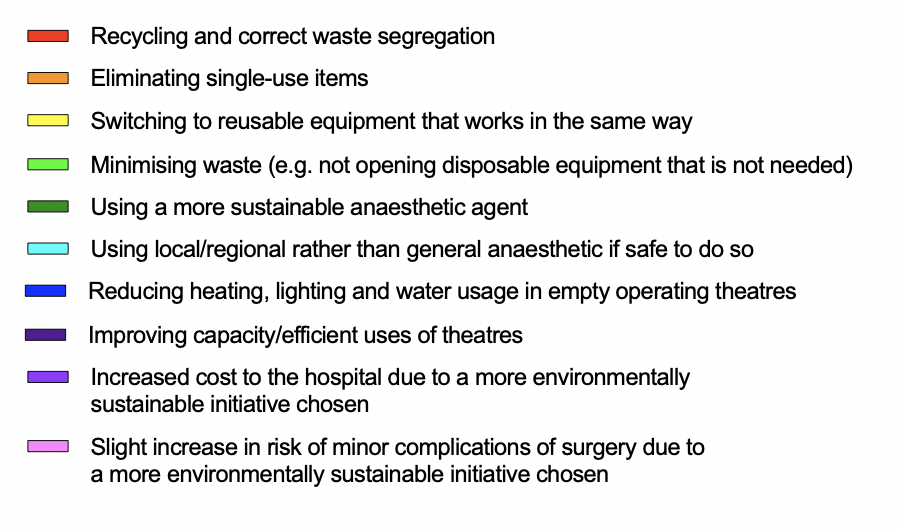
**
